# Initial seronegative immune-mediated necrotising myopathy with subsequent anti-HMGCR antibody development and response to rituximab: case report

**DOI:** 10.1186/s41927-020-00128-5

**Published:** 2020-06-30

**Authors:** Rhys Thomas, Su-Ann Yeoh, Rupert Berkeley, Andrew Woods, Mike Stevens, Silvia Marino, Aleksandar Radunovic

**Affiliations:** 1 Department of Rheumatology, Whipps Cross Hospital, Whipps Cross University Hospital, Barts Health NHS Trust, London, E11 1NR UK; 2 Department of Rheumatology, University College London Hospitals NHS Foundation Trust, London, UK; 3 Department of Radiology, Royal London Hospital, Barts Health NHS Trust, London, UK; 4 Department of Immunology, Oxford University Hospitals NHS Foundation Trust, Oxford, Oxfordshire UK; 5 Department of Pathology, Barts Health NHS Trust, London, UK; 6 Department of Neuropathology, Barts Health NHS Trust, London, UK; 7 Department of Neurology, Royal London Hospital, Barts Health NHS Trust, London, UK

**Keywords:** Anti-HMGCR, Immune-mediated necrotizing myopathy, Myalgia, Case report

## Abstract

**Background:**

Immune-mediated necrotising myopathy (IMNM) is characterised by severe muscle weakness and necrosis with a paucity of inflammation on muscle biopsy. Around 60% of cases are associated with antibodies to the signal recognition particle (SRP) or 3-hydroxy-3-methylglutaryl-coenzyme A reductase (HMGCR); the remainder are seronegative. IMNM is more treatment resistant than inflammatory myopathies.

**Case presentation:**

A 69-year-old woman with previous statin exposure presented aged 63 with muscle weakness and raised creatinine kinase (CK). Anti-SRP and anti-HMGCR antibodies were not detected, but muscle biopsy revealed changes consistent with necrotising myopathy. Statins were discontinued, and she was treated with prednisolone and methotrexate achieving disease remission. Clinical and biochemical parameters were largely stable until 6 years after diagnosis she experienced a rapid deterioration. This was found to be associated with new development of anti-HMGCR antibody. Rituximab was commenced, resulting rapidly in remission. She has remained in remission since, following 2 cycles of rituximab.

**Conclusions:**

To our knowledge, this is the first reported case of serologically negative IMNM whose subsequent rapid deterioration was associated with development of anti-HMGCR antibody. The response to rituximab and subsequent sustained remission suggests a role for early use of rituximab in aggressive cases of anti-HMGCR myopathy.

## Background

Immune-mediated necrotising myopathy (IMNM) is a subtype of myositis characterised by severe muscle weakness, markedly elevated creatine kinase (CK), and necrosis with a relative paucity of inflammation on muscle biopsy [1]. Marked extra-muscular manifestations are uncommon and should prompt the consideration of an alternate diagnosis.

The ability to classify inflammatory myopathies has improved dramatically with the discovery of myositis-specific and myositis-associated antibodies [2]. Around 60% of cases of IMNN are associated with antibodies to the signal recognition particle (SRP) or 3-hydroxy-3-methylglutaryl-coenzyme A reductase (HMGCR) [3], with the remainder of cases having no currently identifiable antibodies. While more accurate classification could lead to greater efficacy subtype-specific therapy, IMNM tends to be more resistant than inflammatory myositis to both conventional and non-conventional treatment [4, 5].

We present a case of initially seronegative IMNM whose subsequent loss of disease control corresponded with the development of anti-HMGCR antibody. To our knowledge, this has never been described in the literature and re-testing for antibodies is not part of common practice. The development of new antibody prompted a change of treatment in this instance.

## Case presentation

**Fig. 1:**
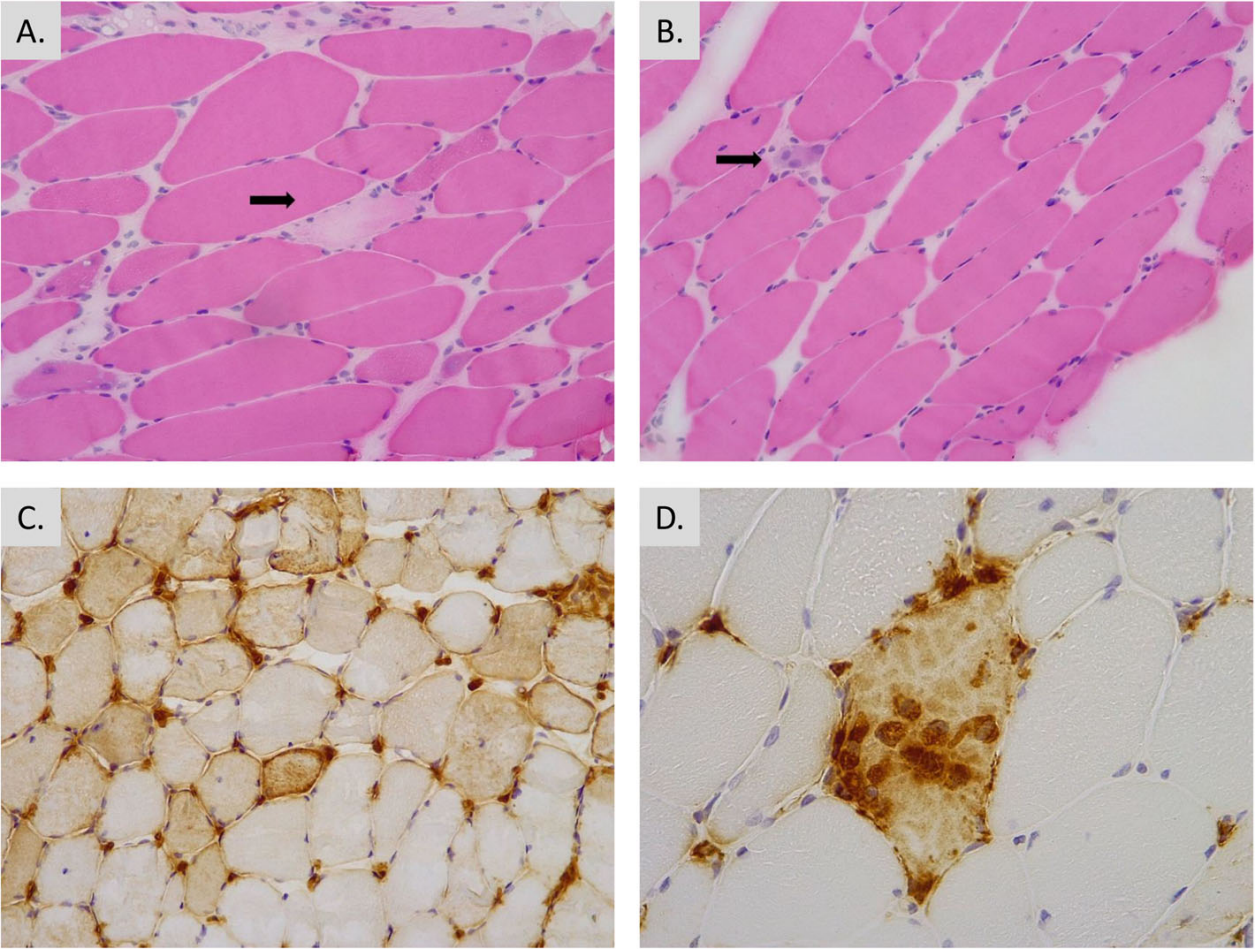
Histopathology from muscle biopsy at time of initial diagnosis. **a** Haematoxylin and eosin stain demonstrating a pale necrotic fibre (arrow). **b** Haematoxylin and eosin stain demonstrating a basophilic regenerating fibre (arrow). **c** MHC class I stain demonstrating patching upregulation of MHC class I. **d** CD68 stain demonstrating a necrotic fibre infiltrated by macrophages

A 69-year Caucasian woman presented at age 63 with progressive upper and lower limb weakness over a 6-month period. Physical examination revealed distal and proximal weakness with no features of extra-muscular disease. Initial creatine kinase (CK) was approximately 6000 iU/L, and single fibre necrosis, predominantly macrophagic inflammation with upregulation of major histocompatibility complex (MHC) Class I at the periphery of fibres and regeneration suggestive of necrotising myopathy was identified on the needle muscle biopsy of the quadriceps muscle (Fig. 1). Autoimmune serology including anti-nuclear antibodies, myositis antibody panel [2] and anti-HMGCR antibody were negative. The presence of anti-HMGCR antibody was assayed for using an in-house developed ELISA method using a commercially available antigen (Sigma-Aldrich) that has been validated against a commercial assay [6].

Malignancy screen including mammogram, computed tomography (CT) body, positron-emission tomography scan and colonoscopy did not suggest any evidence of concurrent malignancy.

Her past medical history included hyperlipidaemia. She commenced 20 mg simvastatin at age 59 but development of myalgia prompted a switch to atorvastatin 40 mg with an initial resolution of symptoms. Statins were permanently discontinued at age 62 by her primary care physician due to return of her symptoms of myalgia.

**Fig. 2:**
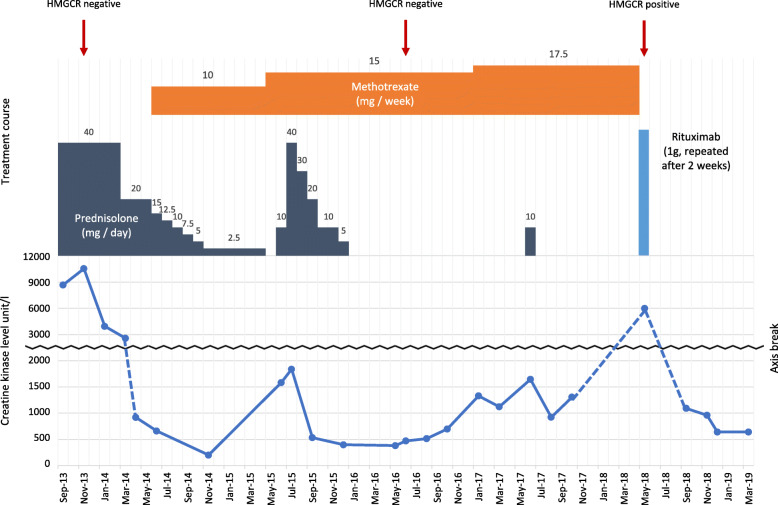
Disease course with creatine kinase levels and treatment

Treatment was commenced 1 year after onset of the symptoms (September 2013) with 40 mg oral prednisolone. This was tapered down over time and stopped 18 months later. Her CK, which had peaked at 10,527 iU/L 15 months from the onset, had fallen to 658 iU/L after 9 months of prednisolone treatment when methotrexate was introduced (Fig. 2).

**Fig. 3:**
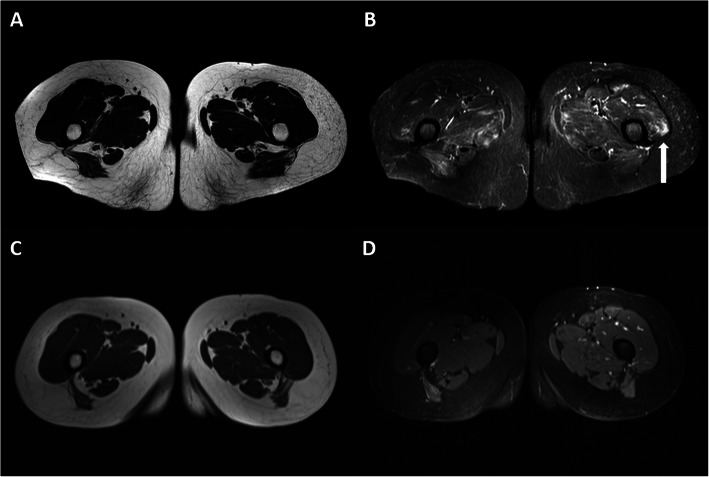
MRI thighs pre- and post-rituximab therapy. Axial **a** T1-weighted FSE and **b** STIR images of the upper thighs in May 2018 showing patchy oedema within the bilateral vasti, adductors and hamstrings. Signal abnormality in the left vastus lateralis is most conspicuous (white arrow). Subsequent axial **c** T1-weighted FSE and **d** STIR images of the same region in March 2019 demonstrate complete resolution of oedema. Muscle bulk remains normal on both studies with no fatty infiltration

During a prolonged period of relative stability, the patient required several weaning courses of prednisolone due to minor clinical and biochemical flares but had remained mostly symptom-free with stable CK levels (Fig. 3). 10 mg per day of prednisolone was re-started due to a rising CK with worsening muscle weakness in June 2015, with methotrexate increased to 15 mg per week. Prednisolone was increased to 40 mg in July 2015 after the patient continued to worsen clinically. This was weaned down by 10 mg per calendar month to 10 mg in November 2015, reducing to 5 mg for December 2015 and stopping in January 2016. HMGCR antibody was retested and was negative in June 2016. Methotrexate was increased to 17.5 mg once weekly in January 2017 due to an asymptomatic rise in CK. She was given 10 mg prednisolone for 1 month in June 2017 to treat a clinical deterioration to good effect. While she largely remained in remission clinically, her CK levels remained raised at a low level suggestive of a partial biochemical remission.

Six years from the onset (May 2018), the patient experienced rapidly worsening proximal weakness with deteriorating mobility and a rising CK to approximately 6000. Magnetic resonance imaging (MRI) of muscles confirmed significant oedema (Fig. 3, panel A & B). A repeat autoimmune screen revealed the presence of anti-HMGCR antibody, detected using the same in-house assay which demonstrated negative results on two previous separate occasions. She received two cycles of rituximab (1 g on day 1 and day 15) 6 months apart. A repeat MRI muscles 3 months after second course of rituximab showed resolution of muscular oedema (Fig. 3, panel C & D), with corresponding clinical and biochemical improvement (CK 600 iU/L). Her anti-HMGCR antibody was re-tested using a commercially available assay (QUANTA Flash, Inova Diagnostics) in December 2019 which confirmed a positive result. The patient remains well and has not required further rituximab therapy. She remains under active review.

## Discussion and conclusions

IMNM remains a rare condition, with very limited randomised control-trial data to guide clinical judgement. The response of anti-HMGCR antibody driven necrotising myositis to rituximab has been mixed in the literature, and this report represents an addition to the evidence base that rituximab is an effective therapy in certain patients with IMNM. Although only describing a single patient, it is important to build a repertoire of treatments that are potentially effective in managing a difficult condition.

Anti-HMGCR IMNM is highly associated with both current and previous statin exposure [7]. However, there is increasing recognition of cases in the paediatric and young-adult population with no prior exposure to statins [8]. Of the 23 anti-HMGCR cases under our care, three have no history of exposure to statins. The pathophysiology of anti-HMGCR IMNM has a clear autoimmune basis and requires long-term immunosuppressive therapy and differs from statin-induced myopathy which typically resolves on withdrawal of the statin [9].

Evidence-based treatment for IMNM is lacking, with treatments derived from case series and clinician experience. The European Neuromuscular Centre working group recommends commencing treatment with corticosteroids plus an alternative agent such as methotrexate [10]. Alternative agents such as azathioprine, mycophenolate mofetil or ciclosporin may be used with limited evidence to suggest superiority of one over any other. Less commonly reported in the literature, rituximab has been used in patients with both anti-HMGCR [11] and anti-SRP positive IMNM [12, 13]. Most reports are in patients who are refractory to treatment with varying treatment responses [14]. The treatment strategy utilised for our patient was to treat early with rituximab at the point of deterioration, bypassing other conventional treatment options (and coincidentally at the point where anti-HMGCR was identified having been previously negative). Rituximab was chosen in this instance before other conventional treatments due to the severity of her relapse, as she was already taking a steroid-sparing agent in methotrexate, and our success with using rituximab in previous IMNM patients. While intravenous immunoglobulin has been demonstrated to be effective in this condition, use of this treatment requires panel approval prior to use in the UK which can introduce delays to patient care. In this patient, use of rituximab has demonstrated ongoing clinical and biochemical effectiveness.

To our knowledge, this is the first reported case of a patient with seronegative, but histology-proven IMNM who subsequently developed anti-HMGCR antibody later in the disease course with associated aggressive trajectory of clinical and biochemical deterioration. Anti-HMGCR antibody has been described to be pathogenic in murine models [15], and a decrease in titre has been shown after successful treatment, although rarely with total normalisation of the antibody levels [7]. The discovery of the development of anti-HMGCR antibody 6 years into the disease course prompted a change in treatment, but current practice is that antibodies are not routinely re-screened for. We suggest that re-screening in the event of a sudden or unexpected clinical change is clinically relevant and may help guide management.

## Data Availability

Not applicable, further information about specifics available on request.

## Abbreviations

IMNM: Immune-mediated necrotising myopathy
SRP: signal recognition particle
HMGCR: 3-hydroxy-3-methylglutaryl-coenzyme A reductase
CK: creatine kinase
MHC: major histocompatibility complex
CT: computerised tomography
MRI: magnetic resonance imaging
